# Roles of N-linked glycosylation and glycan-binding proteins in placentation: trophoblast infiltration, immunomodulation, angiogenesis, and pathophysiology

**DOI:** 10.1042/BST20221406

**Published:** 2023-03-16

**Authors:** Zhengyuan Huang, Pei F. Lai, Alexander T. H. Cocker, Stuart M. Haslam, Anne Dell, Hugh J. M. Brady, Mark R. Johnson

**Affiliations:** 1 Division of Reproductive and Developmental Biology, Department of Metabolism, Digestion and Reproduction, Imperial College London, London SW10 9NH, U.K.; 2 Department of Structural Biology, Stanford University School of Medicine, Stanford, CA 94305, U.S.A.; 3 Department of Life Sciences, Imperial College London, London SW7 2AZ, U.K.

**Keywords:** glycan-binding proteins, hydatidiform mole, immunomodulation, N-linked glycosylation, pre-eclampsia, trophoblast

## Abstract

Protein N-linked glycosylation is a structurally diverse post-translational modification that stores biological information in a larger order of magnitude than other post-translational modifications such as phosphorylation, ubiquitination and acetylation. This gives N-glycosylated proteins a diverse range of properties and allows glyco-codes (glycan-related information) to be deciphered by glycan-binding proteins (GBPs). The intervillous space of the placenta is richly populated with membrane-bound and secreted glycoproteins. Evidence exists to suggest that altering the structural nature of their N-glycans can impact several trophoblast functions, which include those related to interactions with decidual cells. This review summarizes trophoblast-related activities influenced by N-glycan–GBP recognition, exploring how different subtypes of trophoblasts actively adapt to characteristics of the decidualized endometrium through cell-specific expression of N-glycosylated proteins, and how these cells receive decidua-derived signals via N-glycan–GBP interactions. We highlight work on how changes in N-glycosylation relates to the success of trophoblast infiltration, interactions of immunomodulators, and uterine angiogenesis. We also discuss studies that suggest aberrant N-glycosylation of trophoblasts may contribute to the pathogenesis of pregnancy complications (e.g. pre-eclampsia, early spontaneous miscarriages and hydatidiform mole). We propose that a more in-depth understanding of how N-glycosylation shapes trophoblast phenotype during early pregnancy has the potential to improve our approach to predicting, diagnosing and alleviating poor maternal/fetal outcomes associated with placental dysfunction.

## Introduction

Different subtypes of trophoblasts act constitutively to support fetal development *in utero* throughout pregnancy [1,2]. At the molecular level, both trophoblast-derived glycoproteins and glycan-binding proteins (GBPs) that recognize glycans (either as membrane-bound or secreted forms) play roles in cellular invasion, angiogenesis, and immunomodulation [3–7]. Glycoproteins can be N-glycosylated by the covalent attachment of oligosaccharides to the amide nitrogen of Asn located at a Asn-X-Ser/Thr sequence (in which X denotes any amino acid except for Pro). N-glycosylation is initiated in the endoplasmic reticulum (ER) with continued maturation in the Golgi apparatus, of which detailed pathways and kinetics have been illustrated elsewhere [8]. Several N-glycosylated proteins, such as integrins [9], human chorionic gonadotrophin (hCG) [10], human leukocyte antigens (HLAs) [11], pregnancy-specific glycoproteins (PSGs) [12], and immunoglobulin G (IgG) [13] are abundantly expressed at the maternal–fetal interface, and are involved in distinct trophoblastic activities. The role of N-glycan recognition for induction of tolerance during pregnancy has also been supported [14–16]; however, the depth of our understanding for how N-glycosylation is involved in human placental development requires further expansion. This is particularly so regarding differences in the processes of N-glycosylation amongst multiple trophoblast subtypes, and alteration of trophoblastic N-glycomes (the entire N-glycan repertoire [17,18]) in relation to stage of gestation or pathology. In this review, we provide an overview of current knowledge on trophoblast activities that are related to N-glycosylation or N-glycan-GBP recognition (predominantly from on human studies) including observations from trophoblast cell lines, primary trophoblasts and villous tissue explants. Studies using different techniques, such as lectin staining, mass spectrometry, gelatin zymography, and Transwell cell migration/invasion assay, will be highlighted to emphasize the functional relevance of N-glycosylation in trophoblast functions.

## N-glycomes of human trophoblasts

**Figure 1.**
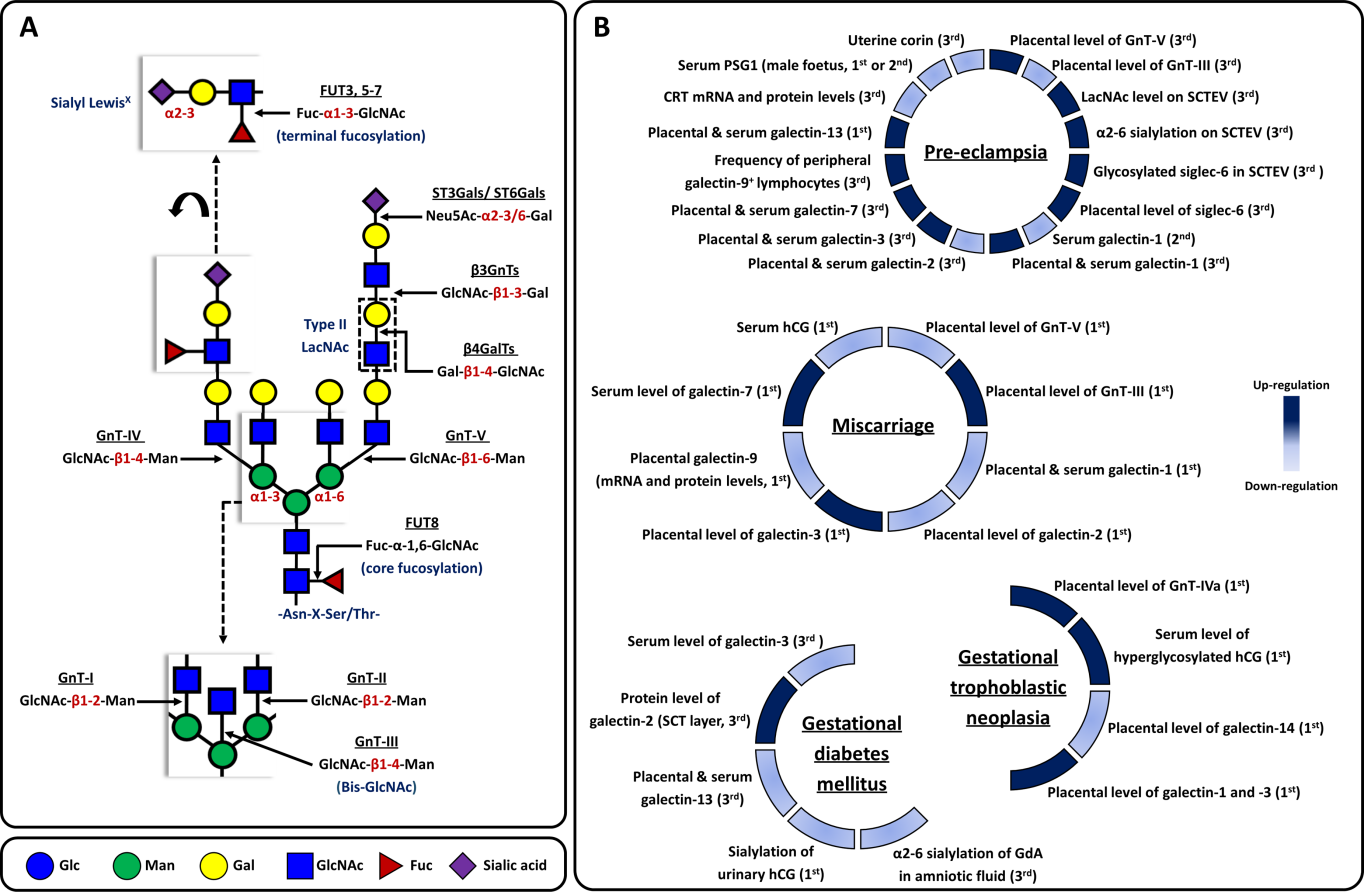
Biosynthesis of N-glycans in eukaryotes and aberrant glyco-codes in representative complications of pregnancy. (**A**) Complex N-glycans are usually modified with extra monosaccharides, which include additional residues attached to the core, additional GlcNAc branches, GlcNAc branches elongated with LacNAc units, and different capping epitopes. Underlined texts indicate glycosyltransferases that catalyze the addition of monosaccharides. Texts in red indicate the glycosidic linkages between two monosaccharides. (**B**) Heatmap based on the relative levels of N-acetylglucosaminyltranseferases (GnTs), galectins, glycoproteins and other factors related to glycosylation, between normal and pathological pregnancies [60,89,101,109,128–136]. The trimester of pregnancy at which each observation was made is given in parentheses.

**Figure 2.**
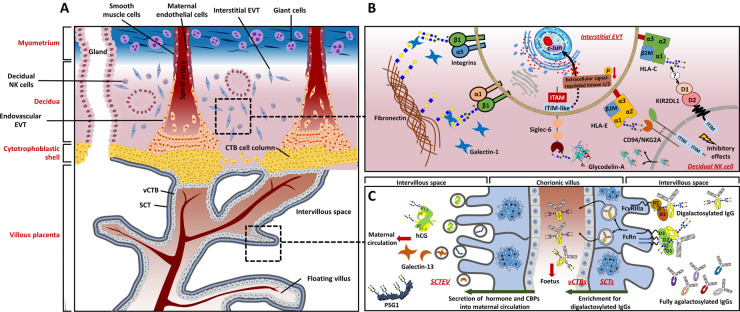
N-glycans and glycan-binding proteins are involved in trophoblast function at the maternal–fetal interface. (**A**) The maternal–fetal interface and trophoblast subtypes. Major cell types contained within the decidua in early first-trimester and associated major trophoblast subtypes are presented. (**B**) Cell–matrix and cell–cell interactions by glycan recognition. Invasive trophoblasts express fibronectin-binding integrin α1β1 and α5β1 to aid migration out from CTB cell column into decidua. To control the invasion of EVTs, decidual NK cells secrete glycodelin-A into the intervillous space that interacts with Siglec-6 expressed by EVTs, which is followed by down-regulation of the extracellular signal-regulated kinase/c-Jun signaling pathway. Although there is evidence highlighting a role for N-glycosylation of HLA Class I molecules in their interaction with inhibitory receptors expressed by decidual NK cells, more details related to this process await elucidation. The depicted structures of N-glycans on β1 integrin [33], glycodelin-A [128], and HLAs [64] correspond to findings from previous studies. (**C**) SCTs secrete vesicles containing hCG or galectin-13 into maternal circulation to promote both decidualization and placentation. At the same time, FcRn and FcγRIIIa expressed on the SCT cell surface collaborate to assist the trans-placental transport of IgGs modified with digalactosylated N-glycans across the villous wall to enter the fetal circulation. The depicted structures of N-glycans on hCG [10], PSG1 [95], and IgGs [13] correspond to findings from previous studies.

Distinct N-glycomes confer the unique phenotypes associated with each trophoblast subtype, which have separate activities at the maternal–fetal interface to support placental development. Both biochemical and mass spectrometric methods have been used for N-glycomic profiling of trophoblasts from different gestational stages of human pregnancy [19–21]. For example, DNA sequencer-aided fluorophore-assisted carbohydrate electrophoresis, complemented by lectin blotting, demonstrated a gestation-related increase in core-fucosylated and multiantennary N-glycans on membrane proteins isolated from placental tissues, but a decrease in biantennary N-glycans with bisecting β1–4 N-acetylglucosamine (Bis-GlcNAc) and α2–3 sialylation (Figure 1A) [19]. Another study used matrix-assisted laser desorption/ionization time-of-flight tandem mass spectrometry to profile the N-glycomes of three primary trophoblast subtypes (Figure 2A), namely villous cytotrophoblasts (vCTBs; isolated from third-trimester placentae), syncytiotrophoblasts (SCTs; obtained after 72 h *in vitro* culture of vCTBs), and extravillous trophoblasts (EVTs; isolated from first-trimester placentae). It concluded that EVTs express relatively lower levels of biantennary N-glycans with Bis-GlcNAc, but higher levels of N-glycans decorated with polylactosamine chains containing repeats of the type II N-acetyllactosamine (LacNAc) unit (Figure 1A) and multiantennary N-glycans, when compared with vCTBs and SCTs. The authors also found that the terminal sialic acids (N-acetylneuraminic acid is the predominant sialic acid found in human cells) from all trophoblast subtypes are primarily in α2–3 linkages [21]. However, it remains to be seen how these findings can be used for the prediction of pregnancy outcomes.

## N-glycosylated proteins and functional GBPs are involved in trophoblast infiltration

### N-glycosylation-related glyco-genes and corresponding glycosyltransferases

Increasing evidence suggests the crucial role of glycosyltransferases in trophoblast migration and invasion, probably achieved via regulating integrin N-glycosylation or trophoblast adhesion to extracellular matrix (ECM) proteins (Table 1) [22–29]. For example, down-regulation of GnT-V expression in JAr (a choriocarcinoma cell line) or HTR-8/SVneo (a transformed human first-trimester trophoblast cell line) not only enhances their invasion and migration, but also reduces the extent of β1–6 GlcNAc branching (product of GnT-V) on α5β1 integrins and increases gelatinolytic activity of matrix metalloproteinase (MMP)-2/9 [22,23]. Furthermore, swainsonine (α-mannosidase class II inhibitor that induces a reduction in the extent of β1–6 GlcNAc branching) treatment also has promoting effects on the migration and invasion of first-trimester villous explants in *in vitro* culture [23]. In contrast, down-regulation of Bis-GlcNAc abundance (Figure 1A) by *MGAT3* (gene encoding GnT-III) silencing inhibits the migration and invasion of HTR-8/SVneo cells [24], which could be attributed to the release of inhibition by Bis-GlcNAc for further processing or elongation of GlcNAc branching [25,26]. The knockdown of the glycosyltransferase responsible for N-glycan core fucosylation (FUT8) also leads to a suppressed migration and invasion in JEG-3 (a choriocarcinoma cell line) and JAr [27]. Overexpression of β1–4 galactosyltransferase 3 (β4GalT3), a glycosyltransferase that adds galactose (Gal) to GlcNAc in a β1–4 linkage to generate type II LacNAc), in HTR-8/SVneo cells and primary human trophoblasts suppresses their migration [28], suggesting that β4GALT3 could play a regulatory role in trophoblast migration.

**Table 1.** **Evidence that glycosyltransferases are involved in trophoblast** **infiltration**

Glycosyltransferases	Ref.	Models	Subjected to	Phenotypes exhibited
GnT-V	[22]	HTR8/SVneo; placental explants	Short hairpin RNA targeting *MGAT5*	↑ Invasion and migration ↑ Gelatinolytic activity of MMP-2/9 ↓ TIMP1/2 expression
				↑ Villous explant outgrowth
	[23]	HTR8/SVneo; villous explant cultures	*In vitro* culture in the presence of swainsonine^1^	↑ Invasion and migration
	[23]	JAr	siRNA targeting *MGAT5*	↑ Adhesion to ECM proteins fibronectin and collagen I/IV ↓ β1–6 GlcNAc branch on α5β1 integrin
GnT-IVa	[32]	JAr	siRNA targeting *MGAT4A*	↓ Migration, invasion and cellular adhesion to ECM proteins fibronectin and collagen I/IV ↓ β1–4 GlcNAc branch on β1 integrin^2^
	[29]	JAr	*MGAT4A* overexpression	↑ Migration, invasion and cellular adhesion to ECM proteins fibronectin and collagen I/IV ↑ Rate of tumor formation in mice
GnT-III	[24]	HTR8/SVneo	siRNA targeting *MGAT3*	↓ Invasion and migration ↓ Gelatinolytic activity of MMP-2/9 ↑ TIMP-1/2 expression^3^
β4GalT3	[28]	HTR8/SVneo; primary EVTs	*B4GALT3* overexpression	↓ Migration (both) and invasion (HTR8/SVneo only) ↑ Adhesion to laminin (HTR8/SVneo only) ↑ Terminal Gal on β1 integrin ↑ Enhances β1 integrin degradation.
FUT8	[27]	JAr and JEG-3	siRNA targeting *FUT8*	↓ Migration, invasion, proliferation and levels of epithelial-mesenchymal transition markers

1 Swainsonine is a potent inhibitor of Golgi α-mannosidase II, which indirectly reduces the generation of β1–6 branch in N-glycans;

2 This conclusion was based on three independent lectin blot analyses of *Datura stramonium* agglutinin binding;

3 TIMP-1/2 are specific endogenous inhibitors of MMP-2/9, which are also expressed in the placenta. Arrows denote up-regulated (↑) or down-regulated (↓) expression compared with the control.

### N-glycosylated proteins in membrane-bound or secreted forms

Characteristic features of the epithelial-mesenchymal transition are observed when vCTBs differentiate, migrate out from CTB cell columns, and invade the decidua [1]. For example, differentiated vCTBs, during movement from the villous space to the placental bed, exhibit reduced levels of laminin-binding α6β4 integrin but increased levels of the fibronectin-binding α1β1 and α5β1 integrins (Figure 2B) [30,31]. Trophoblast-derived integrins are N-glycosylated, whose N-glycomes can be directly influenced by the expression level of glycosyltransferases [23,28,32]. Specifically, the N-glycome of β1 integrin in human placenta has been reported to be gestationally related, as evidenced by differences in electrophoretic mobility between β1 integrins immunoprecipitated from first-trimester (invasive) and term gestation (non-invasive) vCTBs. Treatment of first-trimester β1 integrins with endo-β-galactosidase neutralizes this difference, suggesting that the former contain more polylactosamine chains [33]. Another N-glycosylated protein hCG, one of the gestational hormones abundantly produced and secreted into maternal circulation by SCTs (Figure 2B), is involved in regulating trophoblast invasion [34–37]. Tapia-Pizarro et al. [36] suggested that hCG might alter the endometrial ECM through modulating MMP-2 activity and tissue inhibitor of metalloproteinase 1 (TIMP-1) secretion by primary endometrial stromal cells, which eventually promote the trophoblast invasion. A hCG-isoform characterized by large glycan moieties termed hyperglycosylated hCG (hCG-h), which is produced by invasive CTBs and is the major hCG-isoform found in serum during the first 4–5 weeks of pregnancy [38,39].

Lee et al. [35] compared the stimulatory activities of hCG and hCG-h on trophoblast invasion, and found that hCG-h has a more potent stimulating effect on the invasion of both JEG-3 and primary human trophoblasts than normally glycosylated hCG. These results collectively highlight the potential pro-invasive properties of hCG, yet the mechanism explaining why hCG-h has a more potent stimulating effect awaits further elucidation.

### Calreticulin, Siglec-6, and galectins

N-glycans on endogenous or exogenous glycoproteins are potential ligands for functional GBPs expressed by trophoblasts. Calreticulin (CRT) is a glycan-binding chaperone that aids correct folding of glycopeptides in the ER lumen. ER stress can result in extracellular release of chaperones, and a role in mediating vCTB syncytialization has been recently reported for secreted CRT [40]. A higher level of CRT protein has been observed in first-trimester placenta compared with other gestational periods [41]. Knockdown of CRT expression suppresses invasion of HTR-8/SVneo cells, followed by a weaker attachment to fibronectin that is probably attributed to the markedly changed N-glycan composition on β1 integrin [41]. Heterodimeric α5β1 integrin is a major ligand for fibronectin, and N-glycosylation patterns on both subunits influence their biological functions [42–44]. CRT-overexpressing JEG-3 cells also demonstrate suppressed invasion [45], suggesting that homeostatic control of CRT levels is important for the normal invasive activities of trophoblasts.

Sialic acid-binding immunoglobulin-like lectin 6 (Siglec-6) is a lectin receptor, which preferentially binds to α2–6 sialylated N-acetylgalactosamine, and its placental expression is believed to be specific to humans [46,47]. Siglec-6 is detectable on the surface of TEV-1 (immortalized first-trimester EVT) and JEG-3 cells, acting as a GBP that interacts with α2–6 sialylated N-glycans carried by glycodelin-A (GdA) which is a glycoprotein abundantly produced by the decidua [48]. Significantly, a substantial proportion of the GdA N-glycan antennae are terminated with α2–6 sialylated N-acetylgalactosamine, which is a rare terminal epitope. The Siglec-6/GdA interaction has been proposed to hinder trophoblast invasiveness by reducing extracellular signal-regulated kinase/c-Jun signaling activity (Figure 2B) [49].

The abundance of trophoblast-derived galectins at the maternal–fetal interface highlights their regulatory roles in trophoblast migration and invasion (Figure 2B). The expression of galectins-1, -3, -8, -13, and -14, is developmentally regulated and dependent on trophoblast differentiation. Specifically, galectin-13 (also known as placental protein 13), -14 (placental protein-13-like), and -16 are exclusively expressed by anthropoid primates (including humans) [7,50]. As soluble proteins that are relatively conserved among the lectin family, galectins exhibit high affinity for LacNAc and related structures owing to a unique amino acid sequence motif in their carbohydrate recognition domain. The glycan moiety specificity, functions and sources of expression of galectins restricted to mammalian pregnancy have been reviewed [51]. Studies using primary trophoblasts or cell lines have demonstrated that galectins are involved in the cell–matrix and cell–cell interaction of trophoblasts during placentation (Table 2) [9,52–59]. Among all those placental galectins, galectin-1, -3, -7, and -14 have been related to trophoblast infiltration (migration and/or invasion) via their interactions with ECM proteins according to studies on both invasive trophoblast cell lines and primary EVTs [54,60–62]. Notably, although galectin-8 is selectively expressed by both vCTBs and EVTs at the maternal–fetal interface [63], its functional relevance during pregnancy has not yet been elucidated.

**Table 2.** Evidence that galectins are involved in mediating trophoblastic activities

Galectins	Ref.	Models	Subjected to	Phenotypes exhibited
Galectin-1	[59]	HIPEC-65^1^	Treatment with rhgal-1	↑ Level of membrane-bound HLA-G
			Knockdown of *LGALS1*	↓ Level of membrane-bound HLA-G
	[52]	JEG-3	Treatment with recombinant human IL-2 and TNF-α	↑ Galectin-1 expression
	[55]	BeWo	Treatment with rhgal-1	↓ hCG and progesterone production
	[56]	BeWo and primary CTBs	Treatment with rhgal-1	↑ Cell fusion
	[54]	HTR-8/SVneo and primary CTBs	Treatment with anti-galectin-1 antibody	↓ Invasion
			Treatment with rhgal-1	↑ Invasion
	[57]	Mouse	Knockout of *Lgals1*	↑ Rate of fetal loss in allogeneic mating
Galectin-3	[60]	HIPEC-65^1^	Treatment with rhgal-3	↑ Cell invasion
		SGHPL-4^2^		↑ Number of networks and total length of capillaries
		BeWo		↑ Cell fusion
	[53]	HTR-8/SVneo	Knockdown of *LGALS3*	↓ Migration and invasion, protein level of the β1 integrin, MMP-2/9
	[58]	Mouse	Knockout of *Lgals3*	Impaired differentiation of trophoblast layers
Galectin-7	[61]	HTR-8/SVneo	Treatment with rhgal-7	↑ Adhesion with human endothelial epithelial cells
	[90]	Mouse	Knockout of *Lgals7*	Normal fertility and normal and fertile offspring
Galectin-9	[81]	First-trimester human decidual T cells	Treatment with rhgal-9 and recombinant human IL-27	Differentiation of decidual Tim-3^+^ CD4^+^ T cells into cells with regulatory T cell-like phenotypes
	[82]	Peripheral NK cells	Co-culture with galectin-9^+^ primary CTBs	Transformation into a decidual NK-like phenotype
	[83]	HTR-8/SVneo	Knockdown of *LGALS9*	↑ Susceptibility to NK cytotoxicity
Galectin-13	[108]	Peripheral T cells	Treatment with rhgal-13	↑ Apoptosis and up-regulation of CD95 expression and IL-8 production
	[86]	Peripheral neutrophils		↑ Expression of programed death-ligand 1, hepatocyte growth factor, vascular endothelial growth factor, MMP-9, TNF-α
Galectin-14	[62]	Primary EVTs	Knockdown of *LGALS14*	↓ Migration and invasion, expression of MMP-9 and N-cadherin
Galectin-16	[50]	Peripheral T cells	Treatment with rhgal-16	↑ Apoptosis of CD3^+^ T cells

1 HIPEC-65 is a human invasive, proliferative extravillous cytotrophoblast cell line derived from purified primary EVTs isolated from first-trimester human chorionic villi;

2 SGHPL-4 cells are derived from primary human first-trimester EVT transfected with the early region of SV40 and retain similar invasive potential of normal EVT. Arrows denote up-regulated (↑) or down-regulated (↓) expression compared with the control.

## N-glycosylated immunomodulators and galectins

In mammals, there exists membrane-bound proteins associated with the immune system that are N-glycosylated (e.g. HLA, Toll-like, or T-cell receptors), and some have multiple N-glycosylation sites [64–66]. Preconditioning BeWo cells with glycosyltransferase inhibitors (tunicamycin and castanospermine) prior to co-incubation with peripheral blood-derived CD56^bright^ natural killer (NK) cells can decrease interferon-γ production by the latter, which suggests potential roles for N-glycosylation in modulation of NK cell-mediated inflammatory signaling [15]. Through actively producing transforming growth factor β1 (immunomodulators with several N-glycosylation sites), primary endovascular EVTs isolated from term placenta can promote differentiation of peripheral CD4^+^ T cells (from healthy non-pregnant women) into regulatory T cells with immunosuppressive characteristics, while interstitial EVTs and decidual endothelial cells cannot [67]. The complex and delicate cross-talk between EVTs and decidual immune cells are required in the establishment and maintenance of maternal–fetal immunotolerance; to what extent N-glycosylation influences the immunomodulation of EVTs under physiological conditions remains relatively unexplored.

### HLAs

EVTs have an unusual profile of class I HLA (HLA-I) proteins compared with HLA-null villous trophoblasts, which is a defining feature of EVT differentiation characterized by co-expression of HLA-C (classic HLA-I), and HLA-E, -F, and -G (non-classic HLA-I) [1]. HLA-I variants share a highly conserved N-glycosylation site on their heavy chain subunit [64], and the function of this N-glycosylation site has been related to the correct folding of HLA-Cw1 in ER with the involvement of lectin-like chaperones calnexin and CRT [68].

721.221 (transformed human B lymphocyte) transfectants that express HLA-Cw4 or -Cw6, along with their respective non-glycosylated mutants, have demonstrated that ablation of the N-glycosylation site in HLA-C reduced their recognition by killer cell immunoglobulin-like receptor 2DL1 (KIR2DL1), as well as KIR2DL1-mediated inhibition of NK cell lysis (Figure 2B) [69]. Notably, recent single cell transcriptome analyses demonstrated that three decidual NK cell subsets (namely dNK1, dNK2, and dNK3) express *KIR2DL1* (along with other KIR genes) to different extents; its high expression in the dNK1 subset was confirmed at protein level using flow cytometry and predicted to be indicative of its role in EVT interactions [70]. These observations collectively suggest that N-glycosylation of HLA-C on trophoblasts might mediate NK cell tolerance through KIR2DL1 interaction. In humans, the interaction between specific maternal KIR genotypes and specific fetal/placental HLA-C haplotypes can determine the risk of developing pre-eclampsia [71]. Furthermore, a highly expressed inhibitory heterodimeric receptor on decidual NK cells, the CD94/NKG2A heterodimer, is a C type lectin-like receptor with a conserved carbohydrate recognition domain that specifically binds glycosylated ligand HLA-E expressed by EVTs (Figure 2B) [72]. McMaster et al. [73] have reported that placental HLA-G1 (the isoform that most structurally resembles classic HLA-I) is decorated with complex N-glycans comprising of polylactosamine chains, which is a well-known ligand of major galectins (-1, -3, -8, and -9) at the maternal–fetal interface [74]. The functional relevance of polylactosamine chains on HLA-G to immunosuppressive effects that are possessed by EVTs remains to be established, and methods for obtaining enough endogenous glycoprotein from primary human EVTs to allow more detailed analysis have yet to be developed [4].

### IgG

IgG has a conserved N-glycosylated site (Asn^297^) on each of the heavy chains of its fragment crystallizable (Fc) region [75]. Two neonatal receptors for the Fc region of IgGs, namely neonatal Fc receptor (FcRn) and low affinity IgG gamma Fc region receptor III-A (FcγRIIIa), are co-localized on SCTs and are responsible for receptor-mediated trans-placental transport of IgGs [13,76]. IgG mediates inflammatory activities through the engagement of its Fc with FcγRs and IgGs with different Fc-glycan profiles show distinct affinities for the same FcγR [75,77]. The protein crystal structure of FcγRIIIa-IgG complex suggests that FcγRIIIa has a stronger affinity to afucosylated Fc than fucosylated Fc [78]. Interestingly, an overall increase in galactosylation and sialylation but decreased abundance of Bis-GlcNAc are observed in the Fc-glycan profile of maternal IgG along with advancing gestation [79]. Madeleine et al. [13] reported that maternal IgGs with digalactosylated Fc-glycans and ability to activate fetal NK cells are selectively transferred across the placenta to the neonate because of their enhanced binding to FcRn and FcγRIIIa (Figure 2C), suggesting that antigen-specific Fc-glycan profiles ensure a preferential transfer. However, a more recent study of two clinical cohorts argued that no consistent difference in digalactosylation of maternal and cord IgG1 (the most abundant IgG subclass) and FcRn, rather than FcγRIIIa, contributes to trans-placental transport of IgG [80].

### Galectins exhibiting immunomodulatory effects

Increasing evidence gathered from *in vivo* and *in vitro* studies indicate that lectin–glycan interactions, especially those mediated by the galectin family, are required for immune cell adaptations to tolerate the fetal semi-allograft. Using proliferative EVT cell line HIPEC-65, Tirado-González et al. [59] reported a key role of galectin-1 in modulating HLA-G expression on the trophoblast cell surface, while the exposure of JEG-3 cells to recombinant human IL-2 and tumor necrosis factor α (TNF-α) leads to substantial increase in the expression of galectin-1, together suggesting a putative role of galectin-1 in the prevention of excessive inflammation [52]. The role of trophoblast-derived galectin-9 in the maintenance of immune tolerance during early pregnancy has also been highlighted in human placentae, as evidenced by the induction and promotion of decidual T-cell immunoglobulin and mucin 3 (Tim-3)^+^ CD4^+^ T cells differentiation into cells with Treg-like phenotypes *in vitro* by trophoblast-derived galectin-9 and IL-27 together [81]. Li et al. [82] confirmed that ∼75% of trophoblast cells isolated from first-trimester human placentae are galectin-9^+^ and over 60% of decidual NK cells are Tim-3^+^; through interaction with Tim-3, galectin-9 also induces the transformation of peripheral NK cells into a decidual NK-like phenotype. Moreover, HTR-8/SVneo cells protect themselves from NK-mediated cytolysis by expressing galectin-9 [83]. Unlike galectin-1 and -9 that are expressed by decidual cells, galectin-13 is predominantly expressed by SCTs (localized inside extracellular vesicles, Figure 2C) and, to a lesser extent, trophoblasts next to or within maternal spiral arterioles [84,85]. Galectin-13 is also considered to be immunomodulatory because it induces apoptosis of activated T cells *in vitro*, diverts and kills T cells and macrophages in the maternal decidua, and polarizes neutrophils towards a phenotype facilitating placental growth (Table 2) [86,87]. Galectin-16 has also been demonstrated to induce apoptosis of CD3^+^ T cells; it has been proposed to be the reason why galectin-16 levels increase with greater trophoblastic differentiation (as observed in BeWo and JEG-3 cell lines), whereby more galectin-16 is likely to be needed with advancing gestation to promote immunotolerance that is increasingly required at the maternal–fetal interface [50,88]. Recent progress in understanding the immunomodulatory role of galectins in maternal immune adaption has been summarized elsewhere [50,86,89,90].

## N-glycosylated proteins and GBPs involved in placental angiogenesis

Both hemodynamic and uteroplacental adaptations are required in normal human pregnancy to accommodate the growing fetus, during which N-glycosylated proteins are involved. For example, pregnancy-specific β1-glycoproteins (PSGs) are well-known as pro-angiogenic glycoproteins mainly expressed and secreted into maternal circulation by SCTs (Figure 2C) with advancing gestation [91–93]. Their expression is restricted to species with hemochorial placentation (maternal blood is in direct contact with fetal tissue) [94]. PSG1 is the best studied subtype with highest abundancy. It possesses seven potential N-glycosylation sites of which at least four are substituted with N-glycans whose antennae are potential galectin ligands (complex N-glycan containing polylactosamines chains elongated moieties with mainly α2–3 sialic acid terminals, Figure 1A) [95]. PSG1 not only interacts with α5β1 integrins to modulate EVT adhesion and migration [96], but performs its pro-angiogenic activity and immunomodulatory actions by binding to the latency-associated peptides of anti-inflammatory cytokines transforming growth factor-β1 and -β2, resulting in their activation and increasing the number of regulatory T cells [93,97,98]. Angiogenesis requires the remodeling of ECM through proteases such as MMPs. In particular, the expression of MMP-2 and -9 by EVTs has been highlighted with respect to their ECM-remodeling function [99,100]. PSG1 also induces a higher expression of MMP-2 by HTR8/SVneo cells [93], suggesting a possible mechanism of PSG1-mediated angiogenesis.

Corin is a protease expressed in the human uterus during pregnancy, it has been demonstrated that its expression is reduced in cases of pre-eclampsia [101]. Glycosidase digestion of rat and human corin has been used to show that it is heavily modified with N-glycans [102]. The pro-angiogenic activity of corin has been demonstrated in the activation of atrial natriuretic peptides, which can promote trophoblast invasion and spiral artery remodeling [101]. HEK293 (human embryonic kidney) cells modified to express rat corin have been used to demonstrate the functional necessity of its N-glycans, whereby Asn to Ser substitution mutations at two N-glycosylation sites within the catalytic domain of corin prevented its activation [102]. How reduced levels of corin change the maternal–fetal interface during pre-eclampsia, and whether its glycosylation status affects its function, remain to be established.

Galectin-13 has pro-angiogenic effects and its blood serum concentration increases with advancing gestation in human pregnancy [103,104]. Findings from murine studies have suggested that galectin-13 reduces blood pressure by activating endothelial prostaglandin and nitric oxide signaling pathways [105,106]. For humans, immunohistochemical analysis of placentae obtained from early pregnancy terminations have shown that extracellular galectin-13 tends to aggregate around decidual veins associated with zones of necrosis, containing T cells, neutrophils, and macrophages [84]. It is possible that human SCTs actively secrete galectin-13 into the intervillous space (Figure 2C) during early pregnancy to act as ‘decoys’, by attracting maternal immune cells to minimize their hindrance of invasive trophoblasts during remodeling of maternal spiral arterioles prior to placentation. This decoy mechanism is likely to be specific to anthropoid primates (including humans) because galectin-13 is solely expressed in the hemochorial placenta of these species [50]. Pro-angiogenic roles of galectin-13 and -14 have been highlighted previously, where galectin-13 has been observed to switch peripheral neutrophils towards a phenotype that produces more pro-angiogenic factors that include MMP-9 [86], and knockdown of galectin-14 in primary trophoblasts down-regulates the expression and activity of MMP-9 [62]. Galectin-13 and -14 both induce non-activated T cells to produce the pro-angiogenic cytokine IL-8 [107,108].

## Altered N-glycan–GBP recognition to pathological conditions in pregnancy

Alterations of glyco-codes (e.g. placental N-glycome, proportions of glycoforms on N-glycosylated proteins, or protein levels of GBPs required for trophoblast function) at the maternal–fetal interface have been associated with pathological conditions in pregnancy [16,109–111]. Two studies compared the N-glycome of third-trimester human placenta between uncomplicated pregnancies and those affected by pre-eclampsia using lectin histochemistry: Marini et al. [110] observed a reduction in LacNAc (indicated by *Datura stramonium* agglutinin staining) in the apical border of the SCT, while Sukhikh et al. [112] reported an opposite result for the LacNAc level in SCT using a different LacNAc-binding lectin (*Erythrina Cristagalli* agglutinin). These contradictory observations probably reflect the differing accessibilities of the two lectins to LacNAc ligands in the placental samples. Our understanding of the role of N-glycomic dynamics in pathological pregnancies is still developing. Rigorous physicochemical characterization of trophoblastic N-glycomes is needed in order to assess whether glycosylation changes could be diagnostic of the risk of developing pre-eclampsia. Alternatively, differences between healthy and pathological pregnancies exhibited by N-glycosylation-related enzymes in human placentae (Figure 1B) might be a more feasible approach to aid prediction, diagnosis, and management of clinical complications [85,109,111,113]. For example, an increased level of GnT-V [114] and a decreased level of GnT-III [115] have been observed in term placentae with pre-eclampsia when compared with gestation-matched healthy controls. In contrast, total abundance and enzymatic activity of GnT-V along with the concentration of its product β1–6 GlcNAc in chorionic villi during 6–9 weeks of gestation, are generally lower in placentae from early spontaneous miscarriages compared with those in healthy pregnancies, whereas the reverse was observed for GnT-III and its products [109].

Abnormal N-glycomes of urinary hCG have been related to malignant gestational trophoblastic disease, for example, the N-glycans of hCGs from hydatidiform mole patients contained lower levels of Lewis^X^ antigens (Gal_β1–4_[Fuc_α1–3_]GlcNAc) and higher sialylation than hCGs from healthy early pregnancies [10]. In contrast, most urinary hCGs from choriocarcinoma patients have no sialic acid residues but are more likely to carry tri-antennary N-glycans with a β1–4 GlcNAc branch [116]. Furthermore, different hCG N-glycomes have been found in different stages of pregnancy, sites of synthesis, and other clinical pathologies [4,117]. Guibourdenche et al. used clone B152 antibody raised against a choriocarcinoma-derived hCG to show that only 2–5% of total hCG secreted by primary first-trimester SCT during differentiation are recognized by this antibody. Conversely, 10–20% of total hCG secreted by primary EVTs from the same placentae were recognized despite increased total hCG secretion by SCTs but not EVTs [118]. Thus, correlations between different hCG glycoforms and pregnancy pathologies is of considerable interest.

When considering GBPs, it has been shown that CRT mRNA and protein levels are up-regulated in pre-eclamptic placentae [45]. Siglec-6 has been observed to be more abundant in both the basal plate and chorionic villi of placentae from pre-eclamptic preterm (24–36 weeks of gestation) births, when compared with those from gestation-matched births without pre-eclampsia [119]; an increased level of glycosylated Siglec-6 in SCT-derived extracellular vesicles (SCTEV) from pre-eclamptic placentae has also been reported [120]. The Siglec-6/GdA interaction has been suggested to be involved in the inhibition of trophoblast invasiveness [49]; therefore, the aberrant up-regulation of trophoblast-derived Siglec-6 could be a consequence of abnormal uteroplacental perfusion, which supports the current concepts of pathogenesis of pre-eclampsia characterized by placental ischemia [121].

Aberrant expression of representative galectins in different pathological pregnancies has been summarized; specifically, altered expressions of galectin-1 and -13 in pathological pregnancies, either at placental or serum levels, have been intensively reported [89]. Freitag et al. has shown galectin-1 expression is elevated in placental villi during severe early onset pre-eclampsia (<34 weeks of gestation), when compared with late onset pre-eclampsia (>34 weeks of gestation) and control (normotensive pregnancies, 32–37 weeks of gestation) groups. Thus, maternal serum galectin-1 concentrations at 22 weeks of pregnancy is a potential predictor of pre-eclampsia [113]. Furthermore, spontaneous pre-eclampsia-like syndrome has been observed in wildtype mice during inhibition of galectin-1-mediated angiogenesis [113]. According to four nested case-control studies, serum levels of galectin-13 during the first-trimester are lower in women who subsequently developed preterm pre-eclampsia (delivery before 37 weeks of gestation) compared with those women who had normal delivery at term [122–125]. In contrast, serum levels of galectin-13 during the third-trimester were higher in women presenting with developed preterm pre-eclampsia [126]. This gestational U-turn in serum level of galectin-13 might be attributed to the augmented shedding of galectin-13 into maternal circulation via SCTEV induced by ischemic placental stress (Figure 2C) [85,108,126,127]. Unlike in serum, decreased placental expression of galectin-13 was observed in both miscarriages (first-trimester) and pregnancies presenting with preterm pre-eclampsia (third-trimester) compared with healthy counterparts [108,126].

## Summary

Detailed descriptions of spatiotemporal changes in placental glyco-gene expression and protein levels of glycosidases, glycosyltransferases and GBPs are needed to deduce their role in the modulation of trophoblast function. Furthermore, comprehensive N-glycomic profiling of all human trophoblast subtypes present during early pregnancy would help to determine their relative contributions to placentation and potentially identify novel biomarkers of gestational trophoblastic disease. These data need to be compared with gestation-linked changes in the N-glycosylation patterns of proteins that determine placentation to gain a better insight into their impact on the immunosuppressive activities of trophoblasts in physiological and pathological pregnancies. In all cases, improvements in technology are required to discriminate trophoblast subtypes coupled with stringent clinical phenotyping. Additionally, future studies could focus on the mechanistic aspects of how trophoblast-derived GBPs interact with different cell types in the decidua. These approaches will help to determine: (i) to what extent they contribute to the creation of a uterine microenvironment that promotes healthy placentation and fetal development, and (ii) whether placental glycoproteins can act as therapeutic targets to manage pregnancy complications.

## Perspectives

- Placental N-glycosylation influences immune cell activities at the maternal–fetal interface, which can dictate the progression of pregnancy. Subtle changes at the placental N-glycome caused by disrupted activities of glycosidases and glycosyltransferases in ER and Golgi apparatus, or steric interference at GBPs, have been linked to pathologies that include molar pregnancy, miscarriage, and pre-eclampsia.
- Activities of trophoblasts (e.g. migration and invasion) are dependent on the interaction between GBPs (e.g. Siglec-6 and galectins) with exogenous N-glycosylated proteins (e.g. GdA and integrins), N-glycosylation patterns of several placental proteins (e.g. hCG and HLA), and expression of glyco-genes (e.g. *MGAT3* and *MAGT5*) as demonstrated using siRNA knockdown and pharmacological inhibitors of glycosidase. However, their mechanistic relevance to *in vivo* function needs to be determined to aid evaluation of their use as biomarkers and therapeutic targets.
- The recent development of glycomics has revolutionized how we identify new, as well as re-examine established, glycosylated proteins of interest for a multitude of pathophysiological processes; it is particularly valuable for obtaining more detailed insight for how specific placental N-glycan patterns are linked to different pregnancy complications (by supporting downstream mechanistic studies needed to separate correlation from causation). Demand for its application on complex tissues such as human placenta is ever-present, alongside N-glycan profiling and detection of specific GBPs in maternal blood and urine to predict placental dysfunction in a non-invasive manner to improve reproductive health outcomes.

## Open Access

Open access for this article was enabled by the participation of Imperial College London in an all-inclusive *Read & Publish* agreement with Portland Press and the Biochemical Society under a transformative agreement with JISC.

## Abbreviations

Asn: asparagine
β2M: β2 microglobulin
β3GnT: β1–3 N-acetylglucosaminyltransferase
β4GalT3: β1–4 galactosyltransferase
Bis-GlcNAc: bisecting β1–4 N-acetylglucosamine
CRT: calreticulin
ECM: extracellular matrix
ER: endoplasmic reticulum
EVT: extravillous trophoblast
Fc region: fragment crystallizable region
FcγRIIIa: low affinity IgG gamma Fc region receptor III-A
FcRn: neonatal Fc receptor
Fuc: fucose
FUT: fucosyltransferase
Gal: galactose
GBP: glycan-binding protein
GdA: glycodelin-A
Glc: glucose
GnT: N-acetylglucosaminyltransferase
hCG(-h): (hyperglycosylated) human chorionic gonadotrophin
HLA: human leukocyte antigen
IgG: immunoglobulin G
IL: interleukin
ITI(A)M: immunoreceptor tyrosine-based inhibitory (activating) motif
KIR(2DL1): killer cell immunoglobulin-like receptor (2DL1)
LacNAc: N-acetyllactosamine
Man: mannose
MMP: matrix metalloproteinase
NK: natural killer
PSG: pregnancy-specific β1-glycoprotein
rhgal: recombinant human galectin
Siglec-6: sialic acid binding immunoglobulin-like lectin 6
SCT: syncytiotrophoblast
SCTEV: syncytiotrophoblast-derived extracellular vesicles
Ser: serine
ST3(6)GAL: α2–3(6) sialyltransferase
siRNA: small interfering RNA
Tim-3: T-cell immunoglobulin and mucin 3
TIMP: tissue inhibitor of metalloproteinase
Thr: threonine
TNF-α: tumor necrosis factor α
(v)CTB: (villous) cytotrophoblast
